# Bacterial and fungal infections in COVID-19 patients: A matter of concern

**DOI:** 10.1017/ice.2020.156

**Published:** 2020-04-22

**Authors:** Pengcheng Zhou, Zhenguo Liu, Yuhua Chen, Yinzong Xiao, Xun Huang, Xue-Gong Fan

**Affiliations:** 1 Xiangya Hospital, Central South University, Changsha, China; 2 The Third Xiangya Hospital, Central South University, Changsha, China; 3 Burnet Institute, St Vincent’s Hospital Melbourne, and University of Melbourne, Melbourne, Australia

*To the Editor—*Coronavirus disease 2019 (COVID-19) has become a pandemic. As of April 2, 2020, a total of 896,450 laboratory-confirmed cases have been reported. The death toll from COVID-19 has soared quickly: 45,526 deaths have been reported globally, including 24,692 deaths in only a week (March 26, 2020, through April 2, 2020).^1^ Age, sequential organ failure assessment (SOFA) score, and D-dimer are the main prognostic factors of COVID-19 patients.^2^ The presence of bacterial and/or fungal secondary infection or coinfection is likely another important factor affecting mortality, and it has received inadequate attention.

Bacterial and fungal infections are common complications of viral pneumonia, especially in critically ill patients. They lead to increased need for intensive care and increased mortality. In influenza patients, bacterial coinfection occurs in ~0.5% of healthy young individuals and at least 2.5% of older individuals.^3^ A systematic review revealed that ~1 in 4 H1N1 patients during the 2009 pandemic had a bacterial or fungi infection infection.^4^ Data regarding the bacterial or fungi infection in viral pneumonia led by coronavirus are limited. According to the cohort study report by Zhong Nanshan et al,^5^ 20 of 90 severe acute respiratory syndrome (SARS) patients had secondary lower respiratory tract infections in 2003, which accounted for 70.6% of those critical SARS patients who underwent an invasive operation. The pathogens causing secondary infections in SARS patients were diverse: negative bacilli were the most common but *Candida* was also common.^5^ Invasive pulmonary aspergillosis was another common complication secondary to influenza.^6^

Bacterial and fungal infections in COVID-19 patients have been inadequately investigated and reported thus far. Among the hundreds of articles published with clinical data, only a few have reported secondary infection, mostly without detailed pathogens (Table 1). Even in studies for which secondary infection data are available, the antibiotics use rate (94%–100%) was much higher than the reported incidence of secondary infection (10%–15%).^2,7,8^ In addition, the complication of bacterial or fungal infection was not included in the prognosis analysis in most published papers. Moreover, most of the current infection control protocols aim to prevent the transmission and cross infection by SARS-CoV-2, missing the prevention of bacterial or fungal secondary infection. In fact, secondary infection was found in 50% of nonsurviving COVID-19 patients.^2^

**Table 1.** Secondary Infection or Coinfection in COVID-19 Patients

Patients With Secondary Infection or Coinfection, n/N (%)			Antibiotics Use Rate, n/N (%)			Procalcitonin ≥0.25 ng/mL, n/N (%)			Reference
Total	Nonsurvivors	Survivors	Total	Nonsurvivors	Survivors	Total	Nonsurvivors	Survivors	
28/191 (15)	27/54 (50)	1/137 (1)	181/191 (95)	53/54 (98)	128/137 (93)	20/164 (12)	16/51 (31)	4/113 (4)	Zhou et al^2^
9/52 (17)	4/32 (13)	5/20 (25)	49/52 (94)	30/32 (94)	19/20 (95)	…	…	…	Yang et al^8^
4/41 (10)	4/13 (31)	0/28 (0)	41/41 (10)	13/13 (100)	28/28 (100)	5/39 (13)	3/12 (25)	2/27 (7)	Huang et al^7^

Note. Patients were classified in to ICU and non-ICU patients instead of nonsurvivors and survivors in the study by Huang et al.^7^

Thus far, many diagnostic and prevention approaches to targeting complications in COVID-19 patients have been outlined in clinical guidelines in China. However, little attention has been given to secondary bacterial and fungal infections, and a standardized diagnostic process remains unavailable. A few challenges exist in diagnosing secondary infection in COVID-19 patients. Although it can be difficult to distinguish bacterial or fungal infection and existing viral pneumonia based on clinical and radiological appearance, microbiological examination can add great value to diagnoses, especially sputum culture. However, this approach can pose significant risks to biosample collectors and laboratory technicians processing samples from COVID-19 patients because the virus is transmitted via virus-laden aerosols in addition to respiratory droplets and direct contact.^9^ Thus far, no standardized personal protection equipment (PPE) has been recommended in the guidelines in China for healthcare workers who process bacterial and fungal cultures.^10^ Other problems include insufficient laboratory biosafety conditions and PPE shortages. These conditions have led to most hospitals to decide not to carry out routine microbiological examination in COVID-19 patients, which undermines the diagnosis and treatment of secondary infection.

Clinical data regarding bacterial and fungal infections are valuable in guiding evidence-based treatment of COVID-19. Thus, we call for strengthening the investigation of secondary infection and/or coinfection in COVID-19 patients without risking laboratory staff safety. Health authorities and academic organizations need to include a practical diagnostic process for determining bacterial and fungal infection in COVID-19 patients. Furthermore, the biosafety requirements for COVID-19 microbiological laboratory staff should be issued, and personal protection guidelines for microbiological laboratory staff should be clear. Qualified medical institutions need to be encouraged to carry out necessary microbiological examinations. Thus, we will be able to study bacterial or fungal infections in COVID-19 patients in the following aspects: epidemiology (eg, infection sites, incidence rates, epidemic characteristics, risk factors, etc); pathogens and their drug sensitivity results, thus providing the theoretical and factual evidence for precise treatment; accurate prevention and control of infection complications; and effective reduction of the mortality of COVID-19 patients.
